# Polypill versus medication monotherapy in the prevention of cardiovascular diseases in Iran: An economic evaluation study

**DOI:** 10.1002/hsr2.2240

**Published:** 2024-07-05

**Authors:** Ramin Ravangard, Mohadese Ghanbari, Armin Attar, Abdosaleh Jafari

**Affiliations:** ^1^ Health Human Resources Research Center, School of Management and Medical Information Sciences Shiraz University of Medical Sciences Shiraz Iran; ^2^ School of Management and Medical Information Sciences, Student Research Committee Shiraz University of Medical Sciences Shiraz Iran; ^3^ Department of Cardiovascular Medicine, TAHA clinical trial group Shiraz University of Medical Sciences Shiraz Iran

**Keywords:** cardiovascular diseases, cost‐utility analysis, Iran, Markov model, Polypill

## Abstract

**Background and Aims:**

Cardiovascular diseases (CVDs) are one of the major diseases in developing and developed countries and have high prevalence and mortality rates. Pharmacological interventions, especially the use of combination medications, can have preventive effects in patients with CVDs. Recently, in the PolyIran trial, a combination of atorvastatin, hydrochlorothiazide, aspirin, and valsartan or enalapril (Polypill) was shown to be effective in providing survival benefits as a primary prevention strategy. In the present study, we examine the cost‐effectiveness of the use of polypill compared to its individual components (named as medication monotherapy) in the prevention of CVDs in Iran.

**Methods:**

This was an economic evaluation study conducted to compare the cost‐utility of polypill with that of medication monotherapy for 10,000 hypothetical cohorts of people over 35 years of age using the Markov model and with a lifetime horizon. The study perspective was patient perspective and direct medical costs, quality‐adjusted life‐years (QALYs), and incremental cost‐effectiveness ratio were estimated. To deal with uncertaintysensitivity analyses were used.

**Results:**

The results showed that polypill, with the lowest costs (871 USD) and highest QALYs (14.55), had the most cost‐utility than medication monotherapy. Also, the results showed that the highest sensitivities were related to the utilities of angina and stroke states. At the 21,768 USD threshold, polypill had a 92% probability of being cost‐effective versus other medications.

**Conclusion:**

Considering that polypill had the most cost‐utility, it is suggested that health system policymakers pay special attention to polypill in designing clinical guidelines. Also, through covering this medication by health insurance organizations, it is possible to complete the country's medicine pharmacopeia in preventing CVDs.

## 1 INTRODUCTION

Cardiovascular diseases (CVDs) are a major cause of the global disease burden,^1^ making them one of the leading causes of death and increased healthcare costs.^2^, ^3^ These diseases refer to various conditions that affect the heart or blood vessels and are typically linked to the buildup of fatty deposits in the arteries, known as atherosclerosis, and an elevated risk of blood clots. Additionally, CVDs can cause damage to arteries in vital organs like the brain, heart, kidneys, and eyes.^4^ According to the Atlanta Center for Disease Control and Prevention in 2015, CVDs in the United States kill 61,000 people each year.^5^ In 2016, there were 1.68 million deaths from circulatory system diseases in Europe, accounting for 37.1% of all deaths.^6^ CVDs are also an important cause of death in Asia so that they have led to 10.8 million deaths in 2019.^7^ The majority of deaths from CVDs (87%) are caused by ischemic heart disease (47%) and stroke (40%).^8^ By the year 2030, it is predicted that more than 23.5 million people worldwide will die from CVDs.^9^

In Iran, CVDs are the leading cause of death, particularly in the city of Sari, the country's northernmost region, where the prevalence rate is estimated at 9.2%.^10^, ^11^ The severity of CVDs is steadily increasing in the country.^12^ The main risk factors for CVDs include diabetes, hypertension, dyslipidemia, a family history of heart disease, smoking, physical inactivity, poor diet, and obesity.^13^ Implementing strategies such as prescribing aspirin to high‐risk patients, controlling blood pressure in people with hypertension, managing cholesterol levels, and screening for smoking can effectively reduce the occurrence of CVDs.^14^

Although diet and a healthy lifestyle are important in preventing CVD, they alone are not enough to achieve target levels for LDL‐C, and therapeutic interventions are also needed.^15^, ^16^

Limited resources in low‐ and middle‐income countries may cause difficulties in obtaining cardiovascular (CV) medications.^17^ Some studies indicate that 1–13 times the minimum wage of a worker is spent on buying CV medicines. For this reason, instead of prescribing prophylactic medications based on CVD risk assessment, a more comprehensive approach with a fixed‐dose combination of medications with proven benefits for the prevention of CV disease called polypill, has been proposed.^18^ The use of polypill as a preventive measure for CVDs has the potential to bring several advantages. These include being cost‐effective, safe to use, significantly improving adherence to medication regimens, and enabling better control of risk factors compared to usual care.^19^ However, despite these benefits, the production and distribution of polypill encounter obstacles such as the lack of government reimbursement and low physician uptake.^19^

Another challenge in the case of polypill is the patient's intolerance to the medication, and the side effects of each of its individual components can lead to discontinuation of the medication. However, the side effects of this medication are offset by increased use of and greater adherence to the medication.^20^

Although polypill has many benefits, its harmfulness has also been proven.^21^ The TIPS‐3 clinical trial did not find significant evidence to suggest that the polypill has a positive impact on cognitive and functional decline in people over 65 with CV risk factors.^22^ A study by Sadeghi et al identified primary outcomes of polypill randomized clinical trials, including cardiac death, myocardial infarction (MI), stroke, acute coronary syndrome, revascularization procedures, development or worsening of heart failure (HF), and development of persistent new arterial fibrillation estimated for 34 months.^23^ The polypill generally contains an antiplatelet medication, an antihypertensive medication, and a statin.^24^ The combination of antihypertensive medications targeting multiple mechanisms, such as blocking the renin‐angiotensin system as well as inducing diuresis or vasodilatation, reduces the heterogeneity of the blood pressure responses to a single medication.^25^

PolyIran trial was a two‐group, pragmatic, cluster‐randomized trial. Clusters (villages) were randomly allocated (1:1) to either a package of nonpharmacological preventive interventions alone (minimal care group) or together with a once‐daily polypill tablet (polypill group). This study was the largest clinical trial conducted in the field and showed that the use of polypill was effective in preventing major CV events. Medication adherence was high and adverse event numbers were low. The results showed that the polypill strategy could be considered as an additional effective component in controlling CVDs, especially in LMICs.^26^ In this study, Polypill was a combination of four medications, including aspirin 81 mg, hydrochlorothiazide 12.5 mg, atorvastatin 20 mg, and valsartan 40 mg or enalapril 20 mg.^27^ In the present study, the separate individual components of polypill have been introduced as medication monotherapy, each of which has different uses; aspirin is used to reduce the risk of CV events by 21% and all‐cause mortality by 13% in people with pre‐existing CVD,^28^ Hydrochlorothiazide is the thiazide diuretic for the control of elevated blood pressure. Hydrochlorothiazide acts on the distal convoluted tubules and inhibits the sodium chloride co‐transporter system.^29^ Atorvastatin is used in the primary and secondary prevention of coronary heart disease,^30^ Valsartan and enalapril are angiotensin receptor blockers and angiotensin‐converting enzyme (ACE) inhibitors, respectively. ACE reduces the angiotensin level.^31^

The cost‐utility analysis is a type of economic evaluation studies that consider both the cost and outcome of alternative interventions and the outcome is measured by quality‐adjusted life years (QALYs).^32^

Studies on the cost‐effectiveness, cost, and effectiveness of polypill versus those of polypill individual components have been conducted in countries around the world, which has shown that the use of polypill is more cost‐effective. For example, Gaziano et al. in their study found that the use of polypill to prevent secondary CVDs was cost‐effective.^33^ Barrios et al. concluded in their study that there was a 90.9% probability that polypill was a cost‐effective strategy compared to the use of its separate components (aspirin 100 mg, atorvastatin 20 mg, and ramipril 10 mg).^34^ In Becerra et al.'s study, the probabilistic sensitivity analysis (PSA) showed an 81.5% probability of lower costs of polypill with willingness‐to‐pay £20,000 per QALY gained compared to the costs of its individual components.^35^ Based on a study by Bautista et al, the use of polypill in people at risk for CVDs reduces the risk of lifelong CVDs by 15% in women and 21% in men.^36^ According to a study by van Gils et al, polypill was a cost‐effective strategy for people at higher risk.^37^ In Iran, concerning the effectiveness of polypill, a study has been conducted as a clinical trial, the results of which have shown that polypill compared to the minimum care group, that is, healthy lifestyle training without taking polypill, is more effective in primary and secondary prevention of CVDs.^27^ The cost‐effectiveness of polypill compared to medication monotherapy can assist policymakers in the optimal allocation of financial resources and selecting and implementing cost‐effective interventions for patients with CVDs.

Since researchers could not find a study on the cost‐effectiveness of polypill compared to medication monotherapy in Iran, the present study aimed to determine the cost‐effectiveness of the use of polypill, as a combination medication used in the PolyIran trial, compared to its individual components (i.e., the use of atorvastatin, hydrochlorothiazide, aspirin, and valsartan or enalapril) in the prevention of CVDs in Iran.

## 2 METHODS

**Figure 1:**
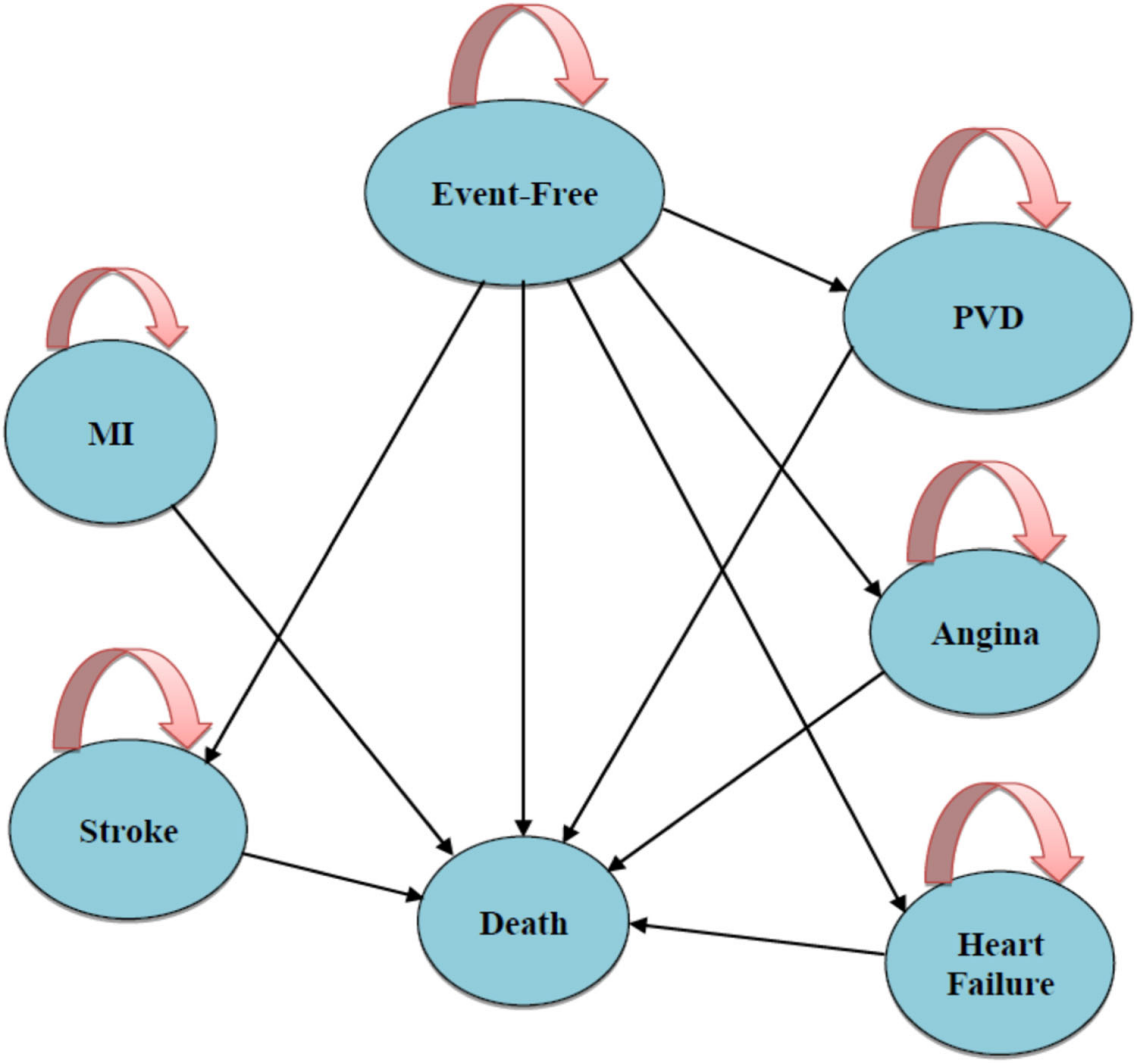
Markov model of cardiovascular diseases. MI, Myocardial infarction; PVD, Peripheral Vascular Disease.

This was an economic evaluation study in which, using the Markov cohort model, the cost‐utility of polypill versus its individual components (i.e., the use of atorvastatin, hydrochlorothiazide, aspirin, and valsartan or enalapril)^27^ in the prevention of CVDs was studied in Iran. The study population in this study included 10,000 hypothetical cohorts of people over 35 years of age^38^ who were distributed among Markov states with annual cycle lengths based on transmission probabilities. These states included event‐free, stroke patients, HF patients, MI patients, angina patients, patients with peripheral vascular disease (PVD), and death. According to this model, people may remain in the event‐free state, enter the states of stroke, HF, MI, angina, PVDs, or die. Patients may also remain in the same states of stroke, HF, MI, angina, PVDs, or death (Figure 1).

In this diagram, people may remain in the event‐free state, enter the states of stroke, HF, MI, angina, PVDs, or die. Patients may also remain in the same states of stroke, HF, MI, angina, PVDs, or death.

The effectiveness index in the present study was the QALYs, which was calculated according to the value of the utility of each of Markov's states (QALY = utility* length of time), extracted from other studies. The lifetime horizon was used in the present study and according to the time horizon, the discount rate was used to determine the current value of costs and outcomes. It is noteworthy that according to internal studies, discount rates of 5.8% was applied for cost, and discount rates of 3% were used for QALYs.^39^

**Figure 2:**
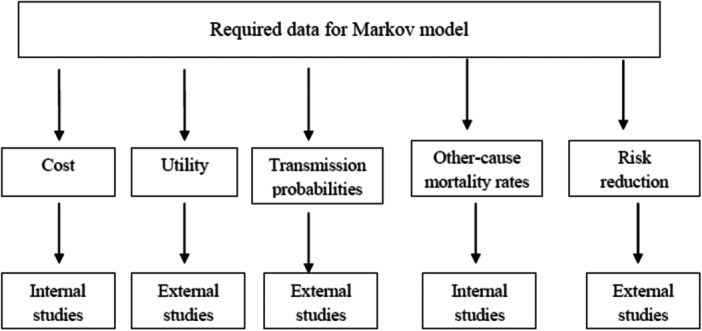
The flowchart of required data for Markov model.

The data required for the study included data on costs, utilities, risk reductions (RR)/hazard ratios (HR), transmission probabilities, probabilities of death in each state, and RRs with statins, which were extracted from the literature (Figure 2) based on the Population, Intervention, comprator, outcome, study design:

Population: CVDs

Intervention: Polypill

Comparator: Medication monotherapy or no intervention

Outcome: Incremental cost per QALY, Cost per death averted, Cost per case averted, rehospitalization, readmission

Study design: Full economic evaluation studies (cost‐effectiveness analysis, cost‐utility analysis, or cost‐benefit analysis), Partial economic evaluation studies.

The perspective in this study was patient perspective, and direct medical costs were taken from national study (Table 1). Due to the lack of internal studies related to the transmission probabilities of MI, angina, and PVDs, RRs with statins, and relative risks (except for stroke and HF in patients taking polypill), they were extracted from international studies. Data related to other‐cause mortality rates were extracted from the life table of Iran (Table 1).^69^ Utility data were also extracted from foreign studies, in which the EQ. 5D questionnaire had been used to calculate utility (Table 1).

**Table 1:** Summary of model inputs.

Variable	Mean	Standard deviation (SD)	Distribution	Source
Probability of stroke	0.5%	0.3%	Beta	[27]
Probability of heart failure (HF)	0.4%	0.23%	Beta	
Probability of myocardial infarction (MI)	1.1−9.4%	0.91−8.7%	Beta	Calculated with Framingham and risk factor profile based on patient‐level data
Probability of angina	1.5−13.3%	1.2−11.3	Beta	
Probability of PVD	0.7−6.2%	0.4−5.4%	Beta	[40, 41]
Utility				
Stroke	0.63	0.56	Beta	[42, 43]
HF	0.68	0.49	Beta	
Acute MI	0.76	0.65	Beta	
Post MI	0.88	0.74	Beta	
Acute Angina	0.77	0.65	Beta	
Post Angina	0.88	0.64	Beta	
PVD	0.9	0.7	Beta	
Event Free	1	1	Beta	[44, 45]
Costs (USD)				
Heart failure (HF)				
Medications	136.7	112	Gamma	[46]
inpatient Costs	128.9	105	Gamma	
Outpatient Costs	1.4	0.8	Gamma	
Average costs of hospitalization for HF	153.0	134	Gamma	[47]
Average costs of HF	419.9	351.8	Gamma	
Stroke				
Average costs of stroke	1215.5	1350	Gamma	[48]
Angina				
Cardiac stress test	9.4	5.1	Gamma	[49]
Electrocardiography	13.6	9.7	Gamma	
Angiography	299.1	189	Gamma	
Visit	48.3	24.2	Gamma	
Average costs of Angina	370.4	228	Gamma	
Myocardial infarction (MI)				
Average Direct costs	259.7	185.6	Gamma	[50]
Aspirin	1.6	1.02	Gamma	[51]
Tissue plasminogen activator	11,200.2	8190	Gamma	[52]
Average costs of MI	11,461.6	8376	Gamma	
PVD				
Average costs of PVD	261.94	183	Gamma	[53]
Enalapril (Per 10): Dose 5	14.82	8	Gamma	Asking the pharmacy
Dose 20	24.68	10		
Aspirin (Per 10): Dose 80	17.14	12	Gamma	Asking the pharmacy
Dose 81	8.57	7		
Dose 100	19.71	14		
Atorvastatin (Per 10): Dose 10	14.57	9	Gamma	Asking the pharmacy
Dose 20	21.42	18		
Dose 40	28.28	20		
Hydrochlorothiazide (Per 10): Dose 25	12.42	8	Gamma	Asking the pharmacy
Dose 50	6.77	5		
Polypill‐E (Per 10)	50.71	43	Gamma	Asking the pharmacy
Polypill‐V (Per 10)	42.85	29		
Risk reduction with statins				
Stroke	0.80	0.6	Log‐Normal	[52, 54]
MI, HF, Angina	0.72	0.53	Log‐Normal	
PVD	0.85	0.41	Log‐Normal	[54]
Risk reduction (RR)				
Enalapril	RR			
Stroke	0.77	0.34	Log‐Normal	[55]
MI	0.72	0.45	Log‐Normal	
Angina	0.74	0.48	Log‐Normal	
HF	0.79 (HR)	0.51	Log‐Normal	[56]
PVD	‐			
Aspirin	RR			
HF	0.71	0.61	Log‐Normal	[57]
Stroke	0.76	0.67	Log‐Normal	[58]
MI	1.01	0.97	Log‐Normal	
Angina	1	1	Log‐Normal	
PVD	0.82	0.75	Log‐Normal	[59]
Hydrochlorothiazide	HR			
MI	0.34	0.23	Log‐Normal	[60]
Stroke	0.73	0.64	Log‐Normal	
Angina	0.71	0.63	Log‐Normal	
HF	1.05	0.94	Log‐Normal	[61]
PVD	‐			
Polypill	HR			
Nonfatal Stroke	0.43	0.23	Log‐Normal	[27]
HF	0.83	0.54	Log‐Normal	
Angina	0.77	0.65	Log‐Normal	[62]
MI	0.66	0.53	Log‐Normal	
PVD	‐			
Probability of death from the event				
Fatal stroke	0.19	0.12	Beta	[44]
Fatal MI	0.19−0.36 (Men) 0.23−0.40 (Women)	0.14−0.29 (Men) 0.19−0.34 (Women)	Beta	
Fatal HF	0.17	0.09	Beta	[63]
SMR after Stroke	2.72	1.43	Beta	[64]
SMR after MI	2.68	1.54	Beta	[65]
SMR after HF	2.17	1.98	Beta	[66]
SMR after Angina	2.19	2	Beta	[67]
SMR after PVD	2.44	2.01	Beta	[68]
Disease‐specific death	0.015	0.003	Beta	[27]

Abbreviations: CV, cardiovascular; HR, hazard ratio; MI, myocardial infarction; PVD, peripheral vascular disease; SMR, standardized mortality ratio.

The collected data were entered into TREEAGE PRO 2011 software and the Markov model was drawn. Then, the incremental cost‐effectiveness ratio, which shows the differences in costs and effectiveness between the two interventions of the use of polypill compared to medication monotherapy ($\text{ICER}=\frac{\text{Difference cost}}{\text{Difference QALY}}$), was calculated. To deal with uncertainty, the one‐way sensitivity analysis (tornado diagram) and PSA were used.^70^ The PSA was performed using the Monte Carlo simulation method. The PSA diagram was drawn by assigning the probability distribution to the parameters. A scatter plot was also drawn. In this plot, wherever there is the highest density and higher confidence level, the intervention is more cost‐effective. After performing PSA, the cost‐effectiveness acceptability curve was used to determine the probability of an intervention being cost‐effective in different willingness‐to‐pay. This curve shows the probability of an intervention being cost‐effective in return for willingness to make different payments. The price index and exchange rate were calculated using the information available on the website of the Central Bank of Iran (each US dollar = 42,000 Iranian Rials).^71^ Also, the willingness‐to‐pay threshold was calculated based on three times the GDP per capita (the World Health Organization method), which was equal to 21,768 USD in 2020.^72^

This study was approved by the Ethics Committee of Shiraz University of Medical Sciences (Code: IR.SUMS.REC.1399.1097).

Tree Age pro 2011 software was used for economic analysis.

## 3 RESULTS

**Figure A1:**
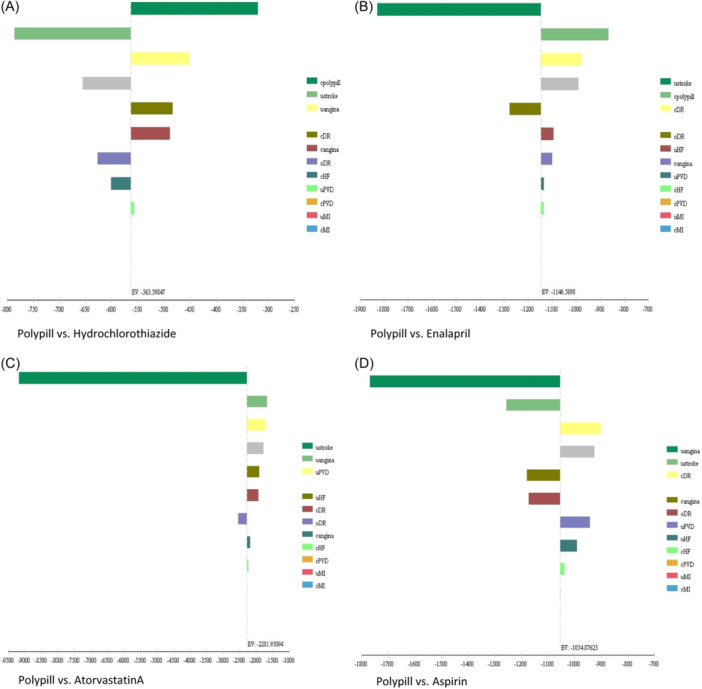
Tornado diagram of the one‐way sensitivity analysis of polypill compared to medication monotherapy. This figure indicated that the results of the study were most sensitive to the utilities of angina and stroke states. (A–D) Polypill had the lowest costs (871 USD) and the highest QALYs (14.55) compared to atorvastatin, hydrochlorothiazide, aspirin, valsartan, and enalapril, and was, therefore, the dominant option.

**Figure A2:**
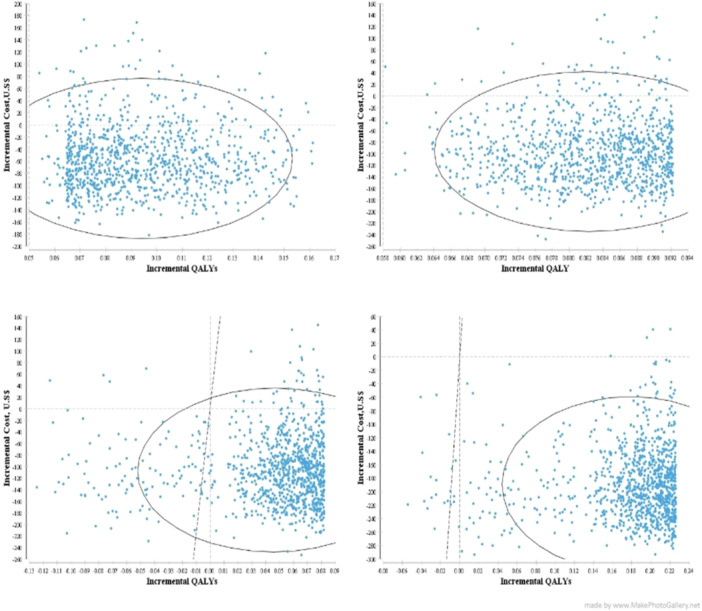
The results of the probabilistic sensitivity analysis of polypill compared to medication monotherapy. The results of Figure 4 indicated that polypill was the dominant option (i.e. it had the lowest cost and highest QALYs) compared to other medications. QALYs, quality‐adjusted life‐years.

The results of the present study showed that among the studied states the MI had the highest costs (11,461.6 USD). Also, the highest and lowest utilities were related to patients in the PVD state (0.9) and patients in the stroke state (0.63), respectively (Table 1). Table A1 shows that polypill had the lowest costs (871 USD) and the highest QALYs (14.55) compared to atorvastatin, hydrochlorothiazide, aspirin, valsartan, and enalapril, and was, therefore, the dominant option. Figure A1 shows the results of one‐way sensitivity analysis (20% increase in parameter value) to compare polypill with other studied medications separately. The results have been shown in the tornado diagram. This diagram showed that the results of the study were most sensitive to the utilities of angina and stroke states. Figure A2 shows the results of PSA for polypill compared to other studied medications. The results of this diagram showed that the distribution of points was mostly located in the southeast quadrant (quadrant 2 of the cost‐effectiveness plan counting clockwise from the north‐east), and therefore, it can be said that polypill was the dominant option (i.e. it had the lowest cost and highest QALYs) compared to other medications. It should be noted that the dashed line indicates the amount of willingness to pay.

**Figure A3:**
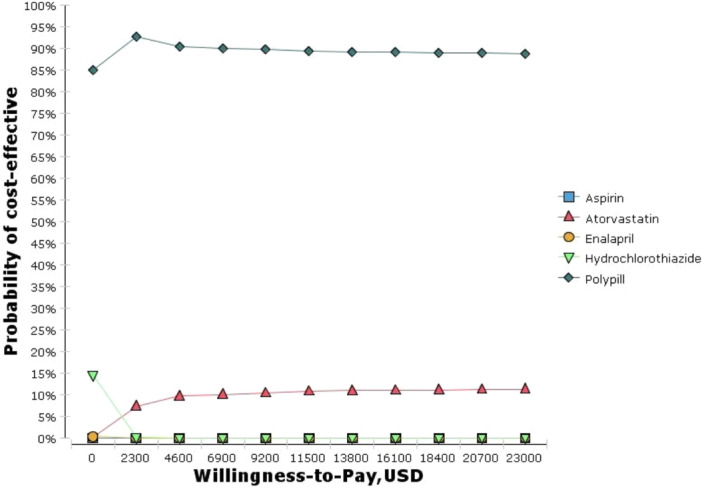
The cost‐effectiveness acceptability curve of polypill compared to medication monotherapy. The results of Figure 5 indicated that at the willingness to pay 21,768 USD, polypill had a 92% probability of being cost‐effective versus other medications.

Figure A3 shows the cost‐effectiveness acceptability curve. The results showed that at the threshold of 21,768 USD, polypill had a 92% probability of being cost‐effective versus other medications.

## 4 DISCUSSION

CVDs are one of the reasons for the loss of health in individuals.^73^ Due to the increasing prevalence and mortality of CVDs worldwide, it will not be possible to achieve the UN Sustainable Development Goal to reduce premature mortality because of CVDs by a third in 2030 for most low‐ and middle‐income countries.^74^ In addition to lifestyle changes, the use of pharmacological interventions is an effective way to accelerate the achievement of this goal. One of the strategies for pharmacological interventions in the prevention of CVDs is to use polypill instead of taking different medications separately. in the current study, we examine the cost‐effectiveness of the use of polypill compared to its individual components (i.e., the use of atorvastatin, hydrochlorothiazide, aspirin, and valsartan or enalapril) in the prevention of CVDs in Iran.

The results of the current study indicated that polypill had the most cost‐utility compared to medication monotherapy, which was confirmed by sensitivity analyses.

Also, the results showed that the average direct medical costs were the highest in patients with MI among the studied states. This could be due to the high costs of medications prescribed for MI patients. The results of the present study are in line with those of the studies by Amirsadri and Sedighi^75^ and Amirsadri and Hassani^76^ in Iran, Ferrante et al. in Argentina,^77^ and Earnshaw et al. in the United States.^78^ However, the results of the present study are not consistent with those of Samuel et al. in Canada,^79^ Jowett et al. in the United Kingdom,^80^ Pignone et al. in the United Kingdom and the United States^58^ which showed that the costs of stroke were higher than those of other states. The reason for this difference can be due to differences in the price of CV disease medications and the amount of insurance coverage in different countries. Moreover, in the current study, the highest utility was related to the PVD state. This may be due to the better mental health status of these patients compared to patients with chronic HF.^81^ In other words, patients with chronic HF are more likely to have end‐of‐life issues because of their advanced disease.^82^ The results of this study are similar to those of Cowie et al.^83^ and Itoga et al.'s^84^ studies, however, inconsistent with those of studies by Amirsadri and Sedighi's study in Iran, in which the utility of MI was the highest,^75^ Earnshaw et al.'s study in the United States, in which the utility of post‐Angina utility was the highest,^78^ Barrios et al. in Spain,^34^ and Becerra et al. (2015) in the United Kingdom,^35^ in which the utility of the nonfatal acute coronary syndrome was the highest among other studied states. One of the reasons for these differences may be the lack of considering the PVD state in these studies.

The results of the present study showed that polypill had the lowest costs (871 USD) compared to the studied medication monotherapy, which is confirmed by the results of the Singh et al.'s study in India.^85^ However, the results of studies by Gaziano et al.^33^ in the United States,^28^ and Jowett et al.^80^ and Becerra et al.^35^ in the United Kingdom, which showed that the highest total costs were related to polypill, are not similar to those of the present study. This difference can be due to differences in the price of polypill in different countries, the nature of the costs (total costs or average costs) studied, and the discount rate used for costs.

According to the results of the present study, the highest QALYs were related to polypill, which is in line with the results of studies by Gaziano et al. in the United States,^33^ Becerra et al. in the United Kingdom,^35^ and van Gils et al. in the Netherlands.^37^

The results of incremental cost‐effectiveness in the current study showed that polypill was a cost‐effective strategy and could replace medication monotherapy The results of the present study are consistent with those of Rubio et al. in Portugal,^86^ Singh et al. in India,^85^ Barrios et al. in Spain,^34^ Bautista et al. in Latin America,^36^ van Gils et al. in the Netherlands,^37^ and Wald et al. in the United Kingdom.^87^ However, Ferket et al.^88^ and Jowett et al. in their studies in the United Kingdom^80^ have concluded that if the annual price of polypill is reduced, it will be a cost‐effective strategy. Zomer et al. in their study in Australia^89^ showed that although polypill could be effective in reducing CV events in patients with metabolic syndrome, it was not cost‐effective. However, in high‐risk populations, for whom combination therapy is often prescribed, polypill is likely to be more cost‐effective than the antihypertensive therapy alone or a combination of statins and antihypertensives. The reason for the differences in the results of these studies can be the differences in the price of polypill and also the differences in its individual components in different countries. The findings of sensitivity analyses showed that the results of the Markov model were slightly sensitive to most parameters. Among the parameters, the highest sensitivities were related to the utilities of angina and stroke states. Overall, it can be said that the model and the results were robust with respect to parameter changes, and the generalizability of the results was appropriate.

This study, like other studies, had some limitations. In this study, due to the lack of internal studies related to the utility, transmission possibilities for MI, angina, and PVD states, RRs with statins, relative risks, and mortality rates, the data from international studies were used. Another limitation of this study was the use of the patient perspective. Therefore, direct nonmedical costs and indirect costs were not considered. Thus, it is recommended to use the societal perspective for more detailed studies on the cost‐utility and cost‐effectiveness of polypill.

This research had limitations. Due to the lack of internal studies on utilities, RR/HR, and transmission probabilities, the required data were taken from international studies. For future studies, it will be suggested to incorporate data from patient registries to increase the external validity of the study. Using patient‐level data will provide more information regarding the diverse patient population and clinical practices. Another limitation is that, in the current study, costs were measured from the patient's perspective, and for future studies, it can be suggested that the researchers should use the societal perspective.

## 5 CONCLUSION

The results indicated that polypill had the most cost‐utility compared to medication monotherapy and it seems that in designing clinical guidelines, the use of polypill can be a good alternative to medication monotherapy due to its lowest costs and highest QALYs. The main challenge of polypill in Iran is its lack of insurance coverage, so it is suggested that health insurance organizations should cover polypill to complete the country's medication pharmacopeia in the prevention of CVDs so that these patients can benefit from its use.

Based on the results of the present study, the polypill strategy could be considered as a cost‐effective option in controlling CVDs, especially in developing countries.

The results of this study should be generalized to other countries with caution because of differences in costs, patients' willingness to pay, incidence and prevalence of CVDs, differences in clinical practice guidelines, differences in medication prices, and differences in discount rates and dollar values among different countries

## AUTHOR CONTRIBUTIONS

**Ramin Ravangard**: Funding acquisition; writing—original draft; writing—review and editing. **Mohadese Ghanbari**: Data curation; writing—original draft; writing—review and editing. **Armin Attar**: Writing—original draft. **Abdosaleh Jafari**: Methodology; supervision; validation; writing—original draft; writing—review and editing.

## CONFLICT OF INTEREST STATEMENT

The authors declare no conflict of interest.

## ETHICS STATEMENT

This study was approved by the Ethics Committee of Shiraz University of Medical Sciences (Code: IR.SUMS.REC.1399.1097).

## TRANSPARENCY STATEMENT

The lead author Abdosaleh Jafari affirms that this manuscript is an honest, accurate, and transparent account of the study being reported; that no important aspects of the study have been omitted; and that any discrepancies from the study as planned (and, if relevant, registered) have been explained.

## Data Availability

All related data were included in the manuscript.

## 1

**Table A1:** The results of cost‐utility analysis of polypill compared to medication monotherapy.

Strategy	Cost (USD)	QALYs
Polypill	871	14.55
Hydrochlorothiazide	924	14.45
Enalapril	965	14.47
Atorvastatin	975	14.5
Aspirin	1058	14.37
Incremental cost per QALYs gained		
Polypill versus hydrochlorothiazide	Dominant	
Polypill versus enalapril	Dominant	
Polypill versus atorvastatin	Dominant	
Polypill versus aspirin	Dominant	

Abbreviation: QALYs, quality‐adjusted life years.
